# 2DKD: a toolkit for content-based local image search

**DOI:** 10.1186/s13029-020-0077-1

**Published:** 2020-02-10

**Authors:** Julian S. DeVille, Daisuke Kihara, Atilla Sit

**Affiliations:** 1 Department of Mathematics and Statistics, Eastern Kentucky University, 521 Lancaster Ave., Richmond, 40475 KY USA; 2 Department of Biology Sciences, Purdue University, 249 S Martin Jischke Dr, West Lafayette, 47907 IN USA; 3 Department of Computer Science, Purdue University, 305 N University Street, West Lafayette, 47907 IN USA; 4 Department of Pediatrics, Cincinnati Children’s Hospital Medical Care, University of Cincinnati, Cincinnati, 45229 OH USA

**Keywords:** Local descriptors, Local image retrieval, Shape matching, Subimage search, Krawtchouk polynomials, Cryo-electron microscopy, Digital pathology

## Abstract

**Background:**

Direct comparison of 2D images is computationally inefficient due to the need for translation, rotation, and scaling of the images to evaluate their similarity. In many biological applications, such as digital pathology and cryo-EM, often identifying specific local regions of images is of particular interest. Therefore, finding invariant descriptors that can efficiently retrieve local image patches or subimages becomes necessary.

**Results:**

We present a software package called Two-Dimensional Krawtchouk Descriptors that allows to perform local subimage search in 2D images. The new toolkit uses only a small number of invariant descriptors per image for efficient local image retrieval. This enables querying an image and comparing similar patterns locally across a potentially large database. We show that these descriptors appear to be useful for searching local patterns or small particles in images and demonstrate some test cases that can be helpful for both assembly software developers and their users.

**Conclusions:**

Local image comparison and subimage search can prove cumbersome in both computational complexity and runtime, due to factors such as the rotation, scaling, and translation of the object in question. By using the 2DKD toolkit, relatively few descriptors are developed to describe a given image, and this can be achieved with minimal memory usage.

## Background

Moment-based approaches are very useful for representing biological and medical images as they are pixelized [1] or voxelized data [2–4]. In medical imaging, such as computerized tomography (CT) scan and magnetic resonance imaging (MRI), objects are observed at different viewpoints and local images need to be extracted and examined. In digital pathology, for instance, pathologists are interested in information about specific structures rather than the whole image [5]. Thus, it is necessary to construct moment invariants that do not change by translation, rotation, and scaling and can efficiently retrieve local image patches or subimages.

Here we present the software package 2DKD, twodimensional Krawtchouk descriptors, for local comparison of 2D images. The mathematical formulation of 2DKD was already established in [1], which brings in the following advantages: 1) Krawtchouk polynomials are defined on a discrete space, so the moments derived from them do not carry any error due to discretization. 2) These polynomials are orthogonal; each moment extracts a new feature of the image, where minimum redundancy is critical in their discriminative performance. 3) They are complete with a finite number of functions (equal to the image size), while many other polynomial spaces have infinitely many members. 4) They have the ability to retrieve local image patches by only changing the resolution of reconstruction and using low order moments. 5) The location of the patch can also be controlled by changing two parameters and hence shifting the region-of-interest along each dimension [6]. 6) These moments can be transformed into local descriptors, which are invariant under translation, rotation, and scaling [1].

2DKD also has the potential to be used in cryo-electron microscopy imaging (cryo-EM), in particular, single-particle cryo-EM. This method generates a 3D reconstruction of the structure by combining data from many 2D projection images, in which identical copies of a protein complex are found in different orientations [7]. From the images of fields containing a large number of molecular complexes, individual particles need to be selected manually or by automated algorithms for further image processing. In addition to the acquisition of high-quality projection images of the particles, fast and accurate particle selection is also critical to ensure a high-resolution 3D reconstruction of structures [8]. We test the software 2DKD by applying it to particle selection in a 2D projection image of GroEL complexes obtained using cryo-EM.

The recognition accuracy of 2DKD was tested in [1] and compared to the traditional Hu invariants on two different datasets, a dataset of binary images and another one with gray-scale clip art images. The comparisons were made based on the top-ranked hits, where the Euclidean distance was used as the similarity measure between two descriptor vectors. Overall, 2DKD showed better prediction accuracies than Hu invariants. The descriptors in [1] were only tested up to 4% noise. Here, we introduce a more stable version of 2DKD, which shows tolerance up to 30% noise in the image data.

## Implementation

### Workflow

**Fig. 1:**
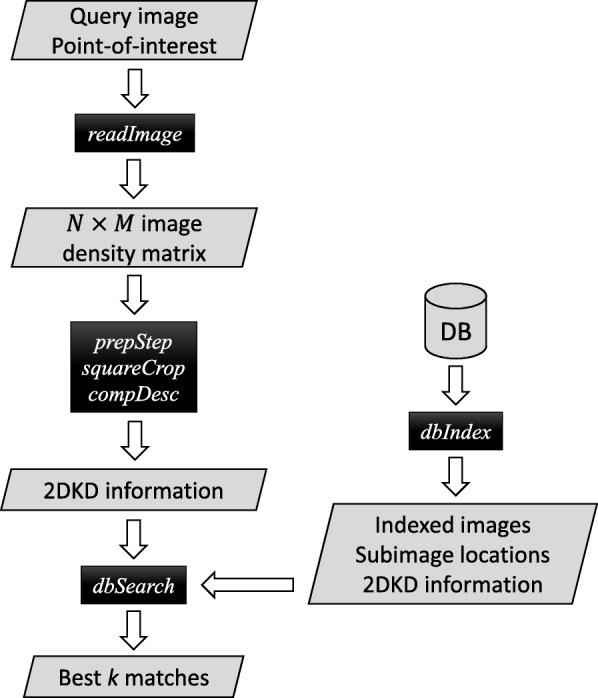
Flow chart of 2DKD. The script names are shown in black boxes
  its geometric moments $\tilde {\mathrm {M}}_{00}$, $\tilde {\mathrm {M}}_{10}$, and $\tilde {\mathrm {M}}_{01}$, the center of mass $(\tilde {x},\tilde {y})$, and the central moments $\tilde {\mu }_{20}$, $\tilde {\mu }_{02}$, and $\tilde {\mu }_{11}$ of $\tilde {f}(x,y)$. It then finds the unique angle $\tilde {\theta }$ between the principal axis of the auxiliary image $\tilde {f}(x,y)$ and the *x*-axis of the 2D plane. This angle is critical for building the rotation invariant descriptors. The exact computation of $\tilde {\theta }$ is provided in [9]. Using $\tilde {\mathrm {M}}_{00}$, $\tilde {x}$, $\tilde {y}$, and $\tilde {\theta }$, this script calculates the geometric invariants $\tilde {\lambda }_{ij}$ for *i*,*j*=0,1,2,3 using the formula provided in [1]. We finally compute the 2DKD using
  2 $$ \begin{aligned} \tilde{Q}_{nm} &= \left[\rho(n;0.5,S-1)\cdot \rho(m;0.5,S-1)\right]^{-1/2}\\ &\cdot \sum_{i=0}^{n}\sum_{j=0}^{m}a_{i,n,0.5,S-1}\cdot a_{j,m,0.5,S-1}\cdot \tilde{\lambda}_{ij} \end{aligned}  $$
  for *n*,*m*=0,1,2,3 and *p*_*x*_=*p*_*y*_=0.5. The descriptors $\tilde {Q}_{00}$, $\tilde {Q}_{01}$, $\tilde {Q}_{10}$, and $\tilde {Q}_{11}$ are removed because they take a constant value irrespective of the region-of-interest we are working with. In this work, we use the 2DKD of order up to 3, that is,

The workflow of 2DKD software is shown in Fig. 1. For a given query image and the pixel location (*x*_*p*_,*y*_*p*_) of a point-of-interest on the image data, 2DKD performs the following six functions.

1. *readImage*: This script reads a standard *N*×*M* gray-scale image file and extracts the image as an *N*×*M* density function *f*(*x*,*y*).
2. *prepStep*: For the number *S* (the size of the query image region) determined by *readImage* or provided by the user, this script computes the 2D central weight function *W*_*c*_(*x*,*y*) corresponding to the parameters *p*_*x*_=*p*_*y*_=0.5 (i.e., the center of an *S*×*S* image). It also computes the norms *ρ*(*n*;*p*,*S*−1) and the coefficients *a*_*i*,*n*,*p*,*S*−1_ corresponding to the Krawtchouk polynomials *K*_*n*_(*x*;*p*,*S*−1) where *n*=0,…,3 and *i*=0,…,*n*. These initial constants computed in prepStep are for later use, so the rest of the computations is performed on-the-fly. A more detailed description of the weight function can be found in [1].
3. *squareCrop*: This script crops an *N*×*M* image density function *f*(*x*,*y*) to a perfect *S*×*S* square image data *f*_*s*_(*x*,*y*). The user-supplied point-of-interest location (*x*_*p*_,*y*_*p*_) in the input image is updated to its relative location (*x*_*s*_,*y*_*s*_) in the square image.
4. *compDesc*: This script translates the central weight function *W*_*c*_(*x*,*y*) to the region of interest within the *S*×*S* square grid as needed. If the local point of interest is located at (*x*_*s*_,*y*_*s*_), then the new weight is defined by *W*_*s*_(*x*,*y*)=*W*_*c*_(*x*^∗^,*y*^∗^) with *x*^∗^=*x*−(*S*−1)/2+*x*_*s*_ and *y*^∗^=*y*−(*S*−1)/2+*y*_*s*_. Whenever (*x*^∗^,*y*^∗^) is situated outside the grid, we set *W*_*s*_(*x*,*y*)=0. The function is defined on the discrete domain {0,1,…,*S*−1}×{0,1,…,*S*−1}. Then using the square *S*×*S* image data *f*_*s*_(*x*,*y*) containing the point (*x*_*s*_,*y*_*s*_), this script first computes the auxiliary (weighted) image
  1 $$ \tilde{f}(x,y) = f_{s}(x,y)\cdot W_{s}(x,y),  $$

3 $$ V = \left[\tilde{Q}_{20}, \,\tilde{Q}_{02}, \,\tilde{Q}_{12}, \,\tilde{Q}_{21}, \tilde{Q}_{30}, \,\tilde{Q}_{03}\right].  $$

Usage example:

- % Change directory to the scripts folder
- >> cd scripts;
- % Full path to the sample image file
- >> impath = ‘../Exp1/DB/image1.jpg’;
- % Point-of-interest location
- >> xp = 180; yp = 480;
- % Read the image to an *N*×*M* density data
- >> [f, S] = readImage(impath);
- % Compute the constants for later use
- >> const = prepStep(S);
- % Crop the image data to a square *S*×*S* data
- >> [fs, xs, ys] = squareCrop(f, xp, yp, S);
- % Compute 2DKD corresponding to (*x*_*p*_,*y*_*p*_)
- >> V = compDesc(fs, xs, ys, const)
- % Output (on the command window)
  V = -0.67263229 -0.67450386 0.00022609 0.00020224 0.00043392 0.00037958

5. *dbIndex*: This high-level script is responsible for producing descriptors for all subimages in the database so that a query can be compared with them. It scans each image in the database by computing the 2DKD for each point-of-interest location and saves the descriptors with the image number and the location of the subimage in that image. The result is stored in a potentially large matrix with rows of the form <Image number, *x*_*p*_, *y*_*p*_, *V*> for easy access later when a subimage is queried. Note that unless there is a change in the database, this only needs to be run once offline to save computational time.
6. *dbSearch*: dbSearch is another high-level script and is used to search the output of *dbIndex* for descriptors similar to the ones corresponding the query. A query image is supplied as input, then *compDesc* is run on the query, producing descriptors for it, and then the matrix from *dbIndex* is sorted by Euclidean distance of descriptors to the new ones obtained, giving a ranked list of the most similar regions to the query from all subimages in the database.

## Results

In this section, we present some experimental results and evaluate the discriminative power of 2DKD. For each point of interest (*x*_*p*_,*y*_*p*_) corresponding to a subimage, we compute and use the feature vector *V* given in (3). To compare the descriptors for a query with those for subimages in a database, we use the squared Euclidean distance as a similarity measure, namely

4 $$ d\left(V^{Q},V^{DB}\right) = \sum_{i=1}^{6} \,\left(V_{i}^{Q}-V_{i}^{DB}\right)^{2}.  $$

### Experiment I

**Fig. 2:**
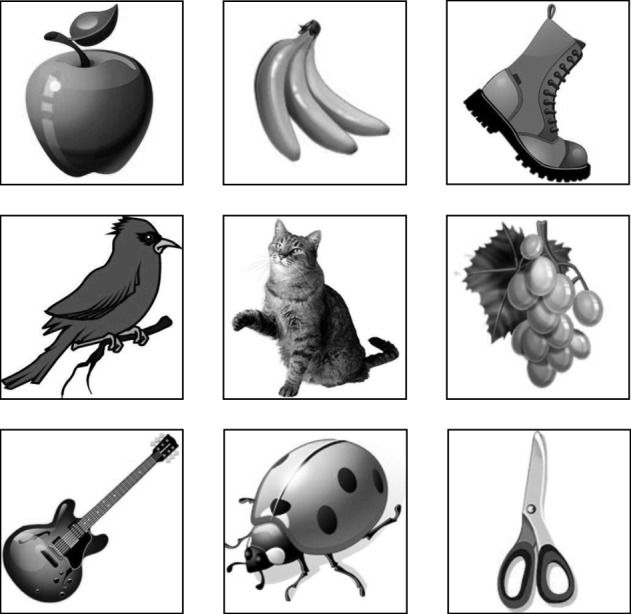
Nine small gray-scale clip art images used as subimages to generate the first image dataset. Image credit: Microsoft Office Online – clip art gallery

**Fig. 3:**
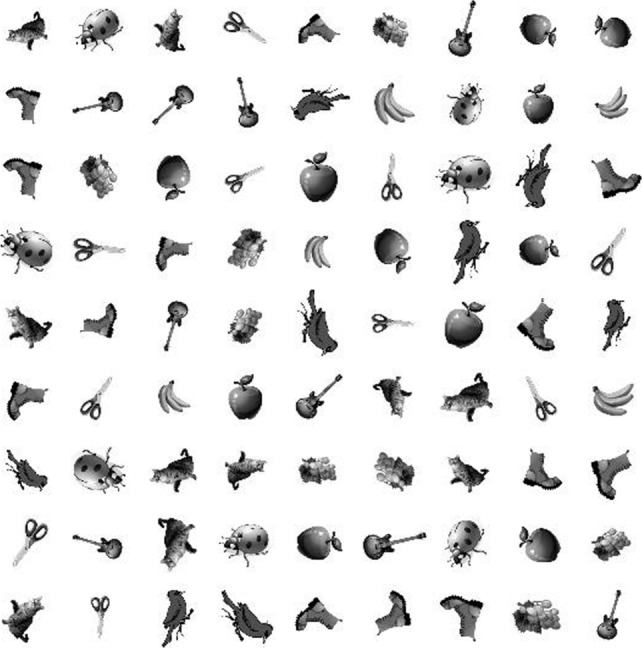
An example 600×600 image from the dataset containing gray-scale clip art subimages

To construct the first database, we use nine clip art icons that are downloaded from Microsoft Office Online. These images are shown in Fig. 2. They are transformed to 60×60 gray-scale images and placed in the center of a 150×150 frame to be used as queries. The same set of gray-scale images are also used to generate a database. These images are rotated by the angles

5 $$ \begin{aligned} \phi = & \;0^{\circ}, 30^{\circ}, 60^{\circ}, 90^{\circ}, 120^{\circ}, 150^{\circ}, \\ & \;180^{\circ}, 210^{\circ}, 240^{\circ}, 270^{\circ}, 300^{\circ}, 330^{\circ}, \end{aligned}  $$

and scaled by the factors

6 $$ \begin{aligned} s = & \;0.8, 0.9, 1.0, \end{aligned}  $$

to obtain a set of 9×12×3=324 subimages. These subimages are randomly placed in 81 positions to form an image of size 600×600. In this experiment, four such images are generated, one of which is shown in Fig. 3.

We run *dbIndex* to produce descriptors for all subimages in the database so a query can be compared with them. After computing the norms, coefficients, and the central weight for *S*=150, *dbIndex* scans each image in the database by trimming a 150×150 region from the image containing each point-of-interest, computes 2DKD for each corresponding subimage, and saves them with the image number and the location of the subimage in that image.

From Table 1, it is clear that 2DKD correctly matches the query subimage with the subimages in the dataset successfully with 100% accuracy if we consider the top-ranked hit and 93.3*%* accuracy when we look at the top 5 hits in the dataset.

**Table 1:** Total hits and misses for 9 queries

Results	Total hits	Total Misses	Accuracy
Top 1	9/9	0/9	100%
Top 5	42/45	3/45	93.3%

**Fig. 4:**
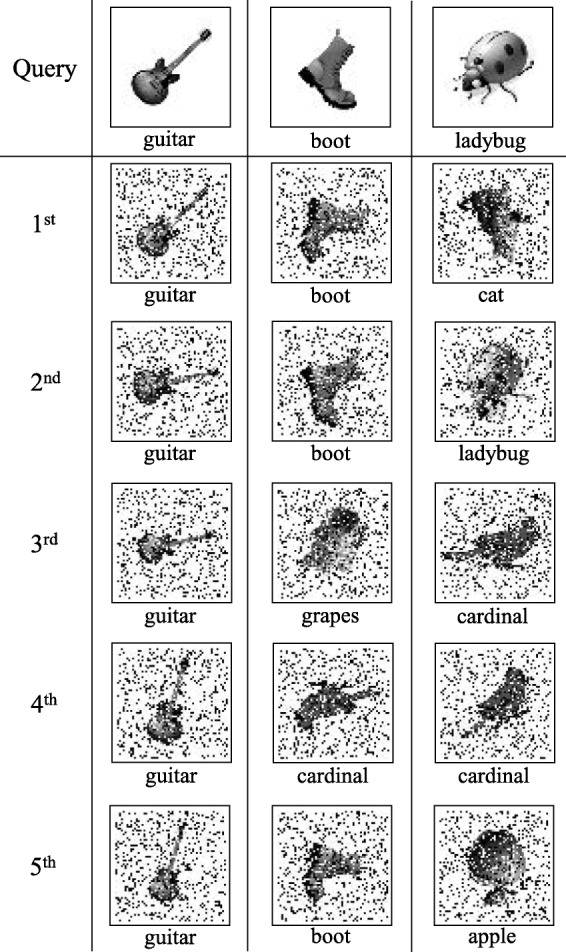
Example queries and corresponding retrievals from the dataset with 30% noise. For each query subimage, top 5 matches from the dataset are shown

We also tested 2DKD for searching for subimages in the salt-and-pepper noise degraded version of the dataset, with noise densities 10%, 20%, and 30%. The results are summarized in Table 2. Considering only top-ranked hits, our descriptors show tolerance up to 30% noise with only one miss. Among top 5 results, it shows 91.1*%* accuracy with 10% noise, whereas it decreases to 77.8*%* with 20% noise, and to 71.1*%* with 30% noise. Three example queries and corresponding top 5 retrievals from the dataset with 30% noise are shown in Fig. 4.

**Table 2:** Total number of successful retrievals from the salt-and-pepper noise degraded dataset

Results	10% noise	20% noise	30% noise
Top 1	9/9 (100%)	9/9 (100%)	8/9 (88.9%)
Top 5	41/45 (91.1%)	35/45 (77.8%)	32/45 (71.1%)

### Experiment II

**Fig. 5:**
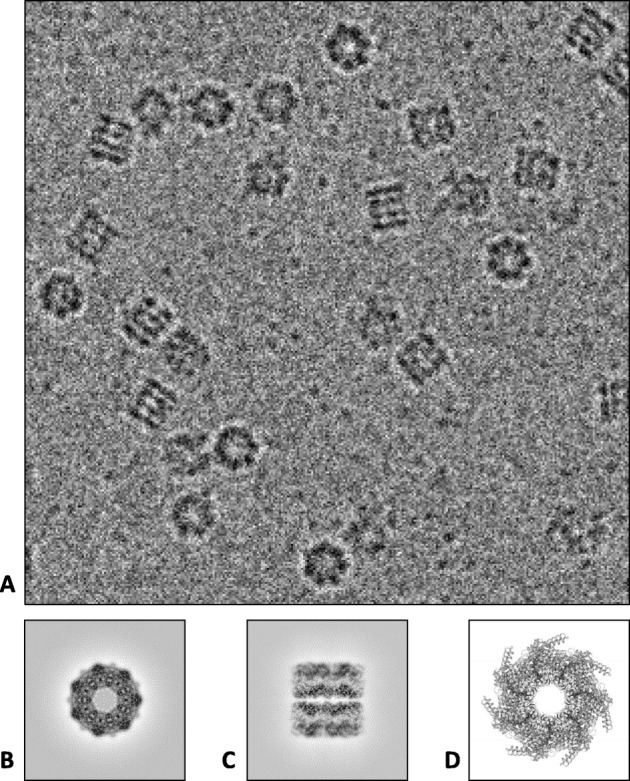
**a** A section of a projection image of GroEL protein complexes in vitreous ice captured using Cryo-EM. **b** Averaged top view of GroEL. **c** Averaged side view of GroEL. **d** An end-on view of the 3D atomic structure of GroEL complex. Image credits – **a** Vossman, https://commons.wikimedia.org/wiki/File:Cryoem\_groel.jpg, **b**, **c**: Electron Microscopy Data Bank (EMD-8750), **d**: Protein Data Bank (PDB ID: 5W0S)

Next, we test the local search performance of 2DKD on a more realistic problem, particle selection in 2D projection images of cryo-EM. In single-particle cryo-EM, these projection images contain identical copies of a protein complex in different orientations. One such example is GroEL, a molecular chaperonin found in a large number of bacteria [10]. An example projection image of GroEL protein complexes is shown in Fig. 5a. From these images, individual particles need to be selected by hand or by automated algorithms. Once selected, they are sorted based on variations on their structural features. Similar images are then averaged to obtain representative projection views of the complex at much higher signal-to-noise ratios than in the original images (see Figs. 5b and c.) Finally, the 3D Fourier transform is built up from a collection of 2D images spanning a complete range of orientations and used to recover the 3D structure of the complex via inverse Fourier transform (see Fig. 5d) [7]. Thus, selection accuracy and speed in particle selection are highly important to increase the resolution of reconstructed 3D structures.

**Fig. 6:**
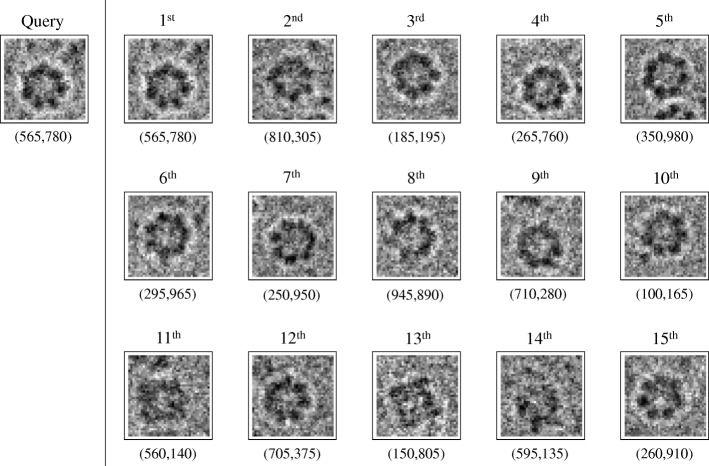
An example query of the top view of GroEL and top 15 retrieval results using 2DKD. The pixel size for the local subimages is 40×40. The (*x*,*y*) centers of the query and retrieval results in the 1024×1024 global image are provided under each subimage

We run the script, *dbIndex*, as in Experiment I to produce descriptors for all subimages in a 1024×1024 projection image (a section of which is shown in Fig. 5a) so a query can be compared with them. The image is very noisy, and there are many flat regions or regions inbetween-particles that should not be considered. We compute the local variance of pixel densities of each 40×40 subregion and compare it against the global pixel density variance. The subregion centers corresponding to lower value of local variances are not indexed. This way, we ensure that we keep the regions with high visibility and discard those with undesired particles. Then we compute 2DKD for each of the remaining subimages using *S*=40 and save them with the (*x*,*y*)−coordinates of the subimage center in the global image. The results are stored in a matrix as in Experiment I. Finally, we query one manually detected top-view of GroEL and search similar ones in the entire indexed image using the script *dbSearch*. The subregions are ranked by the Euclidean distance as in Experiment I, and top 15 hits are demonstrated in Fig. 6. As justified by the figure, most of the retrievals from global image visually match the query except only three of them: the eleventh, thirteenth, and fourteenth. In this experiment, we only search within one image, but the code can be easily adapted to handle a database with multiple projection images.

Table 3 shows the average times taken for computing 2DKD and using them for database indexing and searching. The programs were run 100 times for each task, and the average times were recorded. For each experiment, the programs were tested on a Windows computer with Intel Core i7-8650U processor of 1.90 GHz and 16 GB memory using GNU Octave, version 5.1.0. The table shows that the average time for computing 2DKD of a typical subimage is in the order of 10^−3^, which allows the database indexing to finish in a reasonable amount of time (within a second to under a minute). Assuming that the descriptors were precomputed and stored, the search can be performed in real-time, which makes the software promising for larger datasets.

**Table 3:** CPU times (seconds) for 2DKD

	Experiment I (324 subimages)	Experiment II (11778 subimages)
dbIndex	1.0648	42.7934
Average	3.2865e-3	3.6333e-3
dbSearch	2.6146e-2	9.8097e-2

## Conclusions

Searching biological images for local patterns or specific structures can be computationally challenging due to very low signal-to-noise ratio of these images and the limited number of efficient local invariant descriptors available to perform such searches. We developed 2DKD to address these issues and be used for potentially large biological image databases. 2DKD is developed in Octave (open source) and is publicly available at GitHub website. The source codes can be readily applied to image databases in other fields as well.

## Availability and requirements

**Project name:** 2DKD

**Project home page:** github.com/kiharalab/2DKD

**Operating system:** Windows 7/10, Linux

**Programming language:** GNU Octave (version 5.1.0) or MATLAB R2019a (version 9.6.0)

**Other requirements:** Java (version 8 update 221)

**License:** GNU General Public License (version 3)

## Data Availability

The datasets used in this study are available at the GitHub repository https://github.com/kiharalab/2DKD.

## Abbreviations

2DKD: Two-dimensional Krawtchouk descriptors
Cryo-EM: Cryo-electron microscopy
DB: Database
